# Motivation Types and Mental Health of UK Hospitality Workers

**DOI:** 10.1007/s11469-018-9874-z

**Published:** 2018-01-30

**Authors:** Yasuhiro Kotera, Prateek Adhikari, William Van Gordon

**Affiliations:** Centre for Psychological Research, University of Derby, Kedleston Road, Derby, Derbyshire DE22 1GB UK

**Keywords:** Mental health problems, Mental health attitudes, Work motivation, Internal motivation, Shame, UK hospitality workers

## Abstract

The primary purposes of this study were to (i) assess levels of different types of work motivation in a sample of UK hospitality workers and make a cross-cultural comparison with Chinese counterparts and (ii) identify how work motivation and shame-based attitudes towards mental health explain the variance in mental health problems in UK hospitality workers. One hundred three UK hospitality workers completed self-report measures, and correlation and multiple regression analyses were conducted to identify significant relationships. Findings demonstrate that internal and external motivation levels were higher in UK versus Chinese hospitality workers. Furthermore, external motivation was more significantly associated with shame and mental health problems compared to internal motivation. Motivation accounted for 34–50% of mental health problems. This is the first study to explore the relationship between motivation, shame, and mental health in UK hospitality workers. Findings suggest that augmenting internal motivation may be a novel means of addressing mental health problems in this worker population.

The hospitality industry entails any business offering food, beverages, or accommodation services, including restaurants, pubs, clubs, hotels, contract catering, and hospitality services (Rook 2011). In the UK, the industry is expanding at a faster rate than the economy and employs more than two million workers, which accounts for 7% of the total UK workforce (People 1st 2013a). Hospitality workers are deemed to be one of the most stressed employee populations in the UK. For example, more than 70% of UK hospitality workers report feeling overworked, and 45% take time off due to stress at some point in their career (Davis 2015). Working in the hospitality industry often requires real-time customer interaction along with the ability to provide a quality service (Dann 1990). The requirement to maintain a publicly observable and acceptable emotional display means that hospitality workers can experience distress due to contending with ‘jaycustomer’ behaviour, which refers to thoughtless or discourteous comportment on the part of the customer (Harris and Reynolds 2004). Coupled with the long and anti-social hours worked by many hospitality workers, this ‘emotional labour’ (Hochschild 1985) is a risk factor for depression and other work-related mental health issues (Constanti and Gibbs 2005; Gilmour and Patten 2007).

Distressed workers find it difficult to utilise their creativity fully (Dunnagan et al. 2001) and are more likely to exhibit reduced work effectiveness (Gilmour and Patten 2007). For example, depression hinders productivity and contributes to disability, absenteeism, and possible premature early retirement (Blackmore et al. 2007). Economically speaking, a workplace with distressed workers suffers from a high turnover (International Labour Organization 2010), which yields significant costs to the organisation (Villanueva and Djurkovic 2009). In the UK, more than 10 million working days are lost to stress, depression, and anxiety every year, costing the economy more than £10 billion annually (Paton 2007). The severity of this problem is reflected in the UK hospitality industry (Lashley and Rowson 2000) where the average rate of turnover is 180% for bar staff and over 30% for managers (Badger and Lashley 2000). In terms of monetary implications, the cost of replacing each front-line hospitality employee is approximately £1000, and five times this amount for managers (Lashley and Best 2002).

## Attitudes Towards Mental Health Problems in UK Hospitality Workers

Many UK hospitality workers do not seek professional help for their mental health problems. They exhibit a high degree of shame and are pessimistic about treatment (Kotera et al. 2018). For example, a recent survey indicated that 44% of UK hospitality workers would not talk to anybody if they had a mental health problem (Hospitality and Catering News 2016). In another survey of UK hospitality workers (*n* = 1100), 9% of respondents did not know how to deal with stress, and 38% were afraid to tell their boss that the cause of their absence was work-related stress (Davis 2015). Furthermore, almost 90% of hospitality workers believe susceptibility to stress at work can hinder career progression, and 40% attribute stress to the industry expectation to handle it by themselves (CV-Library 2016). If workers are unwilling to talk about mental illness, they are unlikely to obtain appropriate treatment (Corrigan et al. 2014; Jorm 2012). Therefore, it is unsurprising previous research identified that attitudes and shame towards mental health problems accounted for more than 60% of the variance in mental health problems in UK hospitality workers (Kotera et al. 2018).

## The Role of Work Motivation

Work motivation refers to the psychological factors that induce workers to pursue work-related activities (Pinder 1998). Work motivation has been researched in depth by organisational physicians and scientists, as a highly motivated workforce is a key asset to organisations (Kanfer et al. 2008). Likewise, low levels of work health and motivation are detrimental to organisations and cost £6 billion per year to the UK economy, equivalent of 0.4% of UK GDP in 2012 (Centre for Economics and Business Research 2013).

One of the most established theories underlying work motivation is self-determination theory (SDT), which asserts that human beings have a natural inclination to integrate their psychic elements into a sense of self and larger social structure (Deci and Ryan 1985). SDT separates motivation into intrinsic motivation (i.e. you do an activity because you find it inherently interesting and satisfying) and extrinsic motivation (i.e. you do an activity for an instrumental reason such as money and/or status). According to Ryan’s (1995) model, work motivation can be categorised into six types that, in order of increasing degree of internalisation and autonomy, include (i) amotivation (i.e. where individuals lack the intention to act or act passively), (ii) external regulation (i.e. where individuals do an activity only to obtain a reward [e.g. I work only because I get paid]), (iii) introjected regulation (i.e. where individuals are motivated through self-worth contingencies, such as self-esteem and guilt [e.g. I work because I want people to think I am a good worker]), (iv) identified regulation (i.e. where workers acknowledge the activity’s value and identify with it as their own [e.g. I work because it is important for me]), (v) integrated regulation (i.e. where the value of the activity becomes part of an individual’s sense of self [e.g. I work because I am a hospitality worker; it’s part of who I am]), and (vi) intrinsic motivation (i.e. where individuals work because they find it enjoyable, challenging, and/or a means to actualize their capacity as a human being) (Ryan and Deci 2000). The three lower forms of motivation (i.e. amotivation, external regulation, and introjected regulation) are referred to as non-self-determined motivation or controlled motivation, while the three higher forms of motivation (i.e. identified regulation, integrated regulation, and intrinsic motivation) are classified as self-determined motivation or autonomous motivation (Gagne and Forest 2008; Xu et al. 2014). For the purposes of this paper, controlled motivation or non-self-determined motivation is referred to as ‘external motivation’ (EM) and autonomous motivation or self-determined motivation is referred to as ‘internal motivation’ (IM).

IM is associated with optimal organisational functioning (Gagne and Forest 2011) including active information seeking (Koestner and Losier 2002), goal achievement (Sheldon and Elliot 1999), better work performance (Baard et al. 2004; Miller 2002), well-being (Ilardi et al. 1993), job and life satisfaction (Locke and Latham 2004), and volunteering and prosocial behaviour (Gagne 2003). At an interpersonal level, an autonomy-based work style improves positive work-related outcomes including intrinsic motivation, subordinates’ perceptions, affects (e.g. safe and supported), and job satisfactions such as personal autonomy, job security, and work atmosphere (e.g. Deci et al. 1989; Zuckerman et al. 1978). Work engagement, which implies a positive affective-cognitive state of mind characterised by vigour, dedication, and absorption (Schaufeli et al. 2002), is three times more strongly associated with IM than EM (Cho and Perry 2012). Conversely, the cost of low work engagement is estimated to be approximately $370 billion per year in the USA, equal to 2% of GDP (Gallup 2013). IM can be enhanced by a supportive work climate (Gagne and Deci 2005) and by satisfying the three psychological needs of competence, autonomy, and relatedness (Ryan and Deci 2000).

EM is associated with negative consequences (Vallerand and Ratelle 2002) such as emotional exhaustion, physical and mental health problems (Houkes et al. 2003), depression (Blais et al. 1993), turnover intentions (Quast and Kleinbeck 1990), unstable goal striving (Koestner et al. 1996), susceptibility to persuasion (Koestner and Losier 2002), and compromised performance due to low concentration and memory (Vallerand 1997). Among bank employees and teachers, low work motivation is associated with job burnout and voluntary turnover intentions (Houkes et al. 2001).

In the hospitality field worldwide, the aforementioned types of work motivation and their mental health impacts have been studied to some degree. IM was positively related to job satisfaction and affective organisational commitment, and negatively related to emotional exhaustion among hotel employees in Northern Cyprus (Karatepe and Uludag 2007). A study examining the effects of job resources (e.g. supervision, training, rewards), job demands (role conflict and role ambiguity), and IM on emotional exhaustion in Turkish hotel employees reported that greater job resources and IM led to reduced emotional exhaustion, while greater job demands triggered an increase in emotional exhaustion (Babakus et al. 2008). Furthermore, IM among Nigerian hotel employees was positively related to job performance, and negatively related to emotional exhaustion, emotional dissonance, and turnover intentions (Karatepe and Aleshinloye 2009).

In UK hospitality workers, however, this topic has not been adequately explored. Searches of the electronic search engine EBSCO using the terms ‘UK hospitality workers’ and the aforementioned types of motivation did not yield any relevant academic literature at the time of the current study. The search term ‘UK hospitality workers motivation’ yielded 14 academic papers; however, only one study involving hospitality supervisors/managers in Scotland was directly related to the scope of the this paper. The participants of the Scottish study reported that the principal work-related motivational challenges were low public and self-image of the industry as well as limited career potential and development opportunities (Martin et al. 2006). The remaining 13 unrelated papers included studies focussing on (i) countries outside the UK, (ii) intrinsic motivation as a means of categorising UK seasonal workers (Lee-Ross 1995), (iii) volunteering motivation (Ralston and Rhoden 2005), and (iv) multi-ethnic workgroups (Waser and Johns 2000). Similarly, the words ‘UK hospitality motivation’ yielded 94 academic articles; however, only one article—reporting that IM was associated with more positive experience and higher engagement among students (Hill 2013)—was partially related to the scope of the current study. Searches using other electronic databases yielded one study (Jayaweera 2015) which reported that work motivation (i) mediates a significant relationship between work environment and job performance, and (ii) is significantly associated with job performance in 254 Bristol hotel workers. However, no study has investigated the relationship between types of work motivation and mental health attitudes and problems in UK hospitality workers.

## Study Aims

In light of the aforementioned gap in understanding the relationship between work motivation and mental health attitudes and problems in UK hospitality workers, this study aimed to (i) assess the levels of different types of work motivation in UK hospitality workers, (ii) make a cross-cultural comparison of these motivation levels with Chinese hospitality workers, (iii) investigate the relationship between work motivation, mental health attitudes, and problems, and (iv) identify how work motivation and attitudes towards mental health explain the variance in mental health problems in UK hospitality workers. This study focused on depression, anxiety, and stress as mental health problems because these are the most common types of mental health problems in both the general public and working populations (Davis 2015; European Community 2005).

## Methods

### Participants

UK hospitality workers were recruited using professional networks on social media platforms such as Facebook and LinkedIn. A total of 116 workers agreed to participate of which 103 (47 males, 56 females) completed assessment measures. To be included in the study, participants needed to be aged 18 years or older and have been working for at least 1 year in the UK hospitality industry. Due to the prevalence of part-time work in the UK hospitality industry (People 1st 2013b), both full-time and part-time workers were included (55 full-time, 48 part-time). The age range of participants was 18–55 years (*M* = 28.2, *SD* = 8.6) with 40% working in a hotel, 36% in a restaurant, and the remaining 24% in other hospitality outlets including pubs. The average number of hours worked each week was 46.4 h for full-time workers (*SD* = 8.3) and 20.7 h for part-time workers (*SD* = 6.9). In terms of length of service, 39% of participants had been working in hospitality for more than 5 years, 33% for 2 to 5 years, and the remaining 28% for less than 2 years.

In order to gauge how work motivation in UK hospitality workers compares with hospitality workers in other countries, a comparison was made with Chinese hospitality workers that had previously provided responses to the Work Extrinsic and Intrinsic Motivation Scale (WEIMS (Xu et al. 2014)). Chinese hospitality workers (*n* = 316) were recruited from four- and five-star hotels, based on the Chinese rating system. Of the 316 eligible respondents, 69.6% were female; 49.7% were aged between 16 and 24 years, 32.9% were aged between 25 and 34 years, and the remaining 17.4% were aged 35 years or older. In terms of highest education received, 33.2% of them had high school diplomas, 32.3% had associate degrees, 17.4% were educated to junior high school level or lower, and the remaining 17.1% had a bachelor degree or higher. Typical jobs among these respondents were restaurant server (20.3%), receptionist (19.9%), and room attendant (16.5%). In terms of time in the role, 59.2% of respondents had tenures less than 1 year, 32% had tenures between 1 and 5 years, and the remaining 8.8% had tenures over 5 years (Xu et al. 2014).

### Procedure

Ethical approval was obtained from the university’s research ethics committee. After providing informed consent, participants were sent links to the psychometric scales detailed below. After screening for correctness, UK hospitality workers’ motivation scores were compared with the Chinese counterparts. Correlation analyses were then performed to investigate relationships between motivation types, mental health, and attitudes towards mental health problems. Finally, multiple regression analyses were conducted to examine how much each type of motivation could explain the variance in mental health problems. All analyses were conducted using IBM SPSS version 24.0.

### Measures

#### Work Extrinsic and Intrinsic Motivation Scale

The WEIMS is an 18-item self-report instrument that measures levels of different types of motivations based on SDT (Tremblay et al. 2009). The 18 items comprise three items for each of the following six types of motivation: (i) amotivation, (ii) external regulation, (iii) introjected regulation, (iv) identified regulation, (v) integrated regulation, and (vi) intrinsic motivation. Each item is scored on a seven-point Likert scale (from 1 = ‘Does not correspond at all’ to 7 = ‘Corresponds exactly’). All of the subscales have adequate Cronbach’s alphas of between .64 and .83 (Tremblay et al. 2009).

#### Attitudes Towards Mental Health Problems

The Attitudes Towards Mental Health Problems (ATMHP) is a 35-item self-report instrument measuring attitudes towards mental health problems and three kinds of shame: external, internal, and reflected shame. Attitudes towards mental health problems are assessed by community attitudes (CA) and family attitudes (FA) (i.e. the respondent’s perception of how their community/family perceive mental health problems). External shame is assessed by community external shame (CES) and family external shame (FES) (i.e. the respondent’s perception of how their community/family would perceive them if they had a mental health problem). Internal shame (IS) considers the respondent’s perception of how they would perceive themselves if they had a mental health problem. Reflected shame is assessed by (i) family-reflected shame (FRS) that considers how the respondent believes their family would be perceived if they (i.e. the respondent) had a mental health problem and (ii) self-reflected shame (SRS) that considers how the respondent believes they would feel if a close relative had a mental health problem. Each item is scored on a four-point Likert scale (from 0 being ‘Do not agree at all’ to 3 being ‘Completely agree’). All of the subscales have good Cronbach’s alphas: .85–.97 (Gilbert et al. 2007).

#### Depression Anxiety and Stress Scale

Depression Anxiety and Stress Scale (DASS-21), a 21-item scale, is a short-form version of the DASS-42 (Lovibond and Lovibond 1995), and comprises three subscales that measure depression (e.g. ‘I felt that I had nothing to look forward to’), anxiety (e.g. ‘I felt I was close to panic’), and stress (e.g. ‘I found it difficult to relax’). Participants score how much each statement applied to them in the past week using a four-point Likert scale (0 = ‘Did not apply to me at all’ to 3 = ‘Applied to me very much, or most of the time’). The subscales of DASS-21 have good reliability with Cronbach’s alphas of .94, .87, and .91 for depression, anxiety, and stress, respectively (Antony et al. 1998).

## Results

Table 1 shows descriptive statistics for all outcome measures as well as the difference between UK and Chinese hospitality workers for IM and EM. Three scores in WEIMS and eight scores in ATMHP were identified as outliers using the outlier labelling rule (Hoaglin and Iglewicz 1987) and were thus winsorised (Tukey 1962). Skewness values ranged from − 1.26 to − 0.40, and Kurtosis values from − 1.19 to 1.21. Cronbach’s alpha for all the subscales were above .70, demonstrating high internal consistency. The mean score of depression in our sample of UK hospitality workers was interpreted as severe, anxiety as extremely severe, and stress as moderate-severe (Lovibond and Lovibond 1995).

**Table 1:** Descriptive statistics for all assessment variables and comparison of motivation with Chinese hospitality workers

	UK hospitality (*n* = 103)			Chinese hospitality (*n* = 316)		
Subscale (range)	*M*	*SD*	*α*	*M*	*SD*	*T*
IM (0–7)	5.78^a^	.88	.90	4.51^a^	1.07	10.90
EM (0–7)	5.49^a^	.99	.77	4.11^a^	.71	15.44
CA (0–12)	6.57	2.57	.83			
FA (0–12)	6.33	3.26	.83			
CES (0–15)	9.07	3.51	.85			
FES (0–15)	8.36	4.43	.91			
IS (0–15)	9.34	3.34	.83			
FRS (0–21)	12.06	5.47	.90			
SRS (0–15)	8.30	4.36	.92			
Dep (0–42)	24.36	12.32	.93			
Anx (0–42)	21.94	12.08	.90			
Strs (0–21)	25.78	10.88	.91			

*IM* internal motivation, *EM* external motivation, *CA* community attitudes, *FA* family attitudes, *CES* community external shame, *FES* family external shame, *IS* internal shame, *FRS* family reflected shame, *SRS* self-reflected shame, *Dep* depression, *Anx* anxiety, *Strs* stress

^a^There is a significant difference between the two groups (*P* < .01)

UK hospitality workers had higher levels of both IM and EM compared to Chinese counterparts. The difference in EM was greater between the two groups compared to the difference in IM.

### Correlations

All subscales were square root-transformed to satisfy the assumption of normality. Pearson’s correlations were used to examine relationships between attitude, mental health, and different types of motivation (Table 2). In order to mitigate against the number of correlations calculated, more stringent alpha values were chosen to define statistical significance (alpha = .005).

**Table 2:** Correlations between motivation, shame-based attitudes towards mental health problems, mental health problems, and demographic information

	1	2	3	4	5	6	7	8	9	10	11	GN	Age	WY	WWH
1. IM	.65**	.47**	.42**	.33**	.43**	.47**	.40**	.37**	.41**	.49**	.39**	.10	− .11	.15	.19
2. EM	–	.60**	.65**	.48**	.60**	.47**	.65**	.55**	.76**	.75**	.61**	.25	− .21	.18	.13
3. CA		–	.84**	.71**	.83**	.57**	.73**	.80**	.72**	.74**	.71**	.21	− .14	.19	.13
4. FA			–	.73**	.87**	.51**	.78**	.81**	.81**	.78**	.79**	.25	− .15	.19	.09
5. CES				–	.76**	.59**	.74**	.74**	.59**	.58**	.55**	.19	− .17	.13	− .02
6. FES					–	.62**	.80**	.85**	.73**	.73**	.75**	.20	− .26	.14	.02
7. IS						–	.55**	.45**	.43**	.47**	.42**	.19	− .13	.15	.12
8. FRS							–	.81**	.73**	.74**	.71**	.21	− .24	.18	.07
9. SRS								–	.67**	.70**	.68**	.23	− .23	.20	.09
10. Dep									–	.88**	.87**	.22	− .18	.25	.04
11. Anx										–	.87**	.19	− .15	.20	.06
12. Strs											–	.15	− .14	.23	.02

*IM* internal motivation, *EM* external motivation, *CA* community attitudes, *FA* family attitudes, *CES* community external shame, *FES* family external shame, *IS* internal shame, *FRS* family reflected shame, *SRS* self-reflected shame, *Dep* depression, *Anx* anxiety, *Strs* stress, *GN* gender, *WY* working years, *WWH* weekly working hours

*P < .005; **P < .001

Work motivation, mental health attitudes, and mental health problems were highly related to each other (i.e. all of the combinations were significantly correlated). There was no significant correlation between age, gender, work experience, and weekly working hours. With the exception of internal shame, EM was more strongly related to mental health attitudes and mental health problems than IM.

### Regression

Finally, multiple regression analyses were conducted to explore the relative contribution of the two types of work motivation to depression, anxiety, and stress (Table 3). At step one, gender and age were entered to statistically adjust for their effects, and at step two, the work motivation subscales were entered. Adjusted coefficient of determination (Adj. *R*^2^) was reported. Multicollinearity was not a concern as all of the VIF values were less than 10. After adjusting for demographic information, WEIMS subscales accounted for 50% of the variance for depression and anxiety, and 34% for stress, with EM as a significant explanatory variable.

**Table 3:** Multiple regression: work motivation for depression, anxiety, and stress in UK hospitality workers

	Depression			Anxiety			Stress		
	*B*	SE_*B*_	*β*	*B*	SE_*B*_	*β*	*B*	SE_*B*_	*β*
Step 1									
Gender	.63	.24	.26**	.50	.23	.22*	.35	.20	.18
Age	− .03	.01	− .22*	− .03	.01	− .18	− .02	.01	− .16
Adj. *R*^2^	.08			.05			.03		
Step 2									
Gender	.07	.17	.03	− .01	.16	− .01	− .01	.17	− .002
Age	− .003	.01	− .02	.002	.01	.01	− .001	.01	− .01
IM	− .90	.54	− .14	− .05	.52	− .01	− .06	.54	− .01
EM	4.62	.49	.84**	3.93	.48	.76**	2.71	.50	.61**
Δ Adj. *R*	.50			.50			.34		

*IM* internal motivation, *EM* external motivation, *B* unstandardised regression coefficient, *SE*_*B*_ standard error of the coefficient, *β* standardised coefficient

*P < .05; **P < .01

## Discussion

This study evaluated the levels of two types of work motivation in UK hospitality workers and then made a comparison with Chinese hospitality workers. Following this, an assessment of the relationship between work motivation types, mental health attitudes, and mental health problems was undertaken. Finally, the study investigated the explanatory variables for mental health problems in relation to work motivation.

The current sample of UK hospitality workers scored high on both types of work motivation (i.e. IM and EM). The fact that these scores were comparatively higher than the Chinese hospitality workers could be indicative of a tendency for UK hospitality workers to exhibit a high degree of either internal or external motivation. In other words, internally motivated UK hospitality workers are highly internally motivated, while externally motivated workers are highly externally motivated. The difference in motivation levels between UK and Chinese hospitality workers was much larger for EM. This may relate to the fact that many individuals working in UK hospitality do not perceive their job as a career (Wildes 2007). For example, UK student workers who work at a restaurant part-time are often motivated by external financial factors. In contrast, the Chinese hospitality employees working at four- or five-star hotels—of which 50% were aged 25 years or older, and more than 65% had high school diplomas or associate degrees—are more likely to perceive their job as career. The cross-cultural difference in levels of IM may also relate to the UK hospitality industry’s demanding working conditions that typically involve long and anti-social working hours, low salary, job instability, seasonal employment, and low job status (Wildes 2005).

There were significant correlations between work motivation, mental health attitudes, and mental health problems. With the exception of the internal shame component of mental health attitudes, EM was more strongly related to mental health attitudes and mental health problems compared to IM. The stronger correlation of EM versus IM to mental health problems is consistent with findings from previous studies indicating that EM exerts less of a protective influence over psychopathology versus IM (e.g. Fernet 2013; Raeissi et al. 2014). Furthermore, the correlation between work motivation and mental health attitudes suggests that externally motivated workers tend to feel more shame about having mental health problems compared to internally motivated workers. A plausible explanation for this is that externally motivated workers do not identify with their work and are thus unable to justify work-related mental health problems, whereas internally motivated workers can justify work-related adversity to a greater extent.

Multiple regression analyses revealed that EM was the significant explanatory variable for depression, anxiety, and stress, and that it accounted for 34–50% of the variance in these mental health problems, after adjusting for age and gender. This finding is consistent with studies demonstrating the significance of employee work motivation to mental health in worker populations outside the UK (Babakus et al. 2008; Karatepe and Aleshinloye 2009; Karatepe and Uludag 2007). Furthermore, this study outcome is consistent with the fact that positive motivation-based psychological approaches have been found to be an effective means of alleviating mental health problems (Kobau et al. 2011).

In terms of the implications of these findings, strengthening internal motivation may be an effective means of improving UK hospitality workers’ mental health. Research demonstrates that enhancing mental health awareness is a common approach to addressing mental health problems (e.g. Kline and Sussman 2000); however, it may also be beneficial for organisations to try to augment employee levels of internal motivation through training or other interventions. To provide an example of how this could work in practice, the Disney strategy, a dynamic Neuro-Linguistic Programming skill that considers dreams and plans (Dilts 1998), could seek to identify what workers want internally from their lives and career, by accessing the dreamer, realist, and spoiler positions (Kotera and Sheffield 2017). In this strategy, modelled from how Walt Disney achieved his dreams, clients are requested to (i) dream of their future aspirations as if nothing was impossible (dreamer position), (ii) plan the action steps needed to achieve the dream (realist position), and (iii) search for any gaps in their dream and plans (spoiler position). Participants reported that this strategy was especially useful for identifying what they internally aspired to do in their life and career (Kotera and Sheffield 2017). To a certain extent, this is already being done in the izakaya (Japanese pub) industry of Japan where it is common to have a daily staff meeting at the beginning of work where employees share their dreams and ambitions with each other (e.g. Teppen 2012). The regression analyses conducted in the present study indicate that these types of interventions may have a role to play in enhancing internal motivation as a means of improving employee mental health.

This study was limited by several factors. Firstly, participant recruitment was conducted via opportunity sampling, which hinders the generalisability of the study findings. Secondly, although the comparison with Chinese hospitality workers allows levels of work motivation in UK hospitality workers to be viewed in a more global context, future studies could compare findings with (i) hospitality workers in a greater range of countries and (ii) UK workers in industries other than hospitality. Lastly, the causal direction of these effects has not been investigated. In future studies, longitudinal data would help (i) elucidate the temporal patterning of the observed relationships and (ii) with the development of interventions that would shed light on causality.

Levels of psychological distress and mental illness among employees in the fast-growing UK hospitality industry are concerning. The problem is compounded because due to their attitudes towards mental health, including feeling ashamed, many UK hospitality workers are reticent to seek help. Although organisations are aware of the impacts of motivation on mental health attitudes and problems, this is the first study to explore their relationship in UK hospitality workers. In line with this study’s main finding that external motivation is a significant explanatory variable for mental health problems in UK hospitality workers, intervention studies are warranted to determine whether enhancing internal motivation improves mental health problems in this population group. Considering their working hours, a brief intervention is likely to be a more effective approach.
